# Identification of compound heterozygous patients with primary hyperoxaluria type 1: clinical evaluations and in silico investigations

**DOI:** 10.1186/s12882-017-0719-y

**Published:** 2017-10-02

**Authors:** Houda Kanoun, Faiçal Jarraya, Bayen Maalej, Amina Lahiani, Hichem Mahfoudh, Fatma Makni, Jamil Hachicha, Faiza Fakhfakh

**Affiliations:** 1 Laboratoire de Génétique Moléculaire Humaine, Faculté de Médecine de Sfax, Université de Sfax, Sfax, Tunisie; 2 Unité de Recherche Pathologie rénale UR12ES14 et Service de Néphrologie, Hôpital Hédi Chaker Sfax, Sfax, Tunisia; 3 Service de Pédiatrie, Hôpital Hédi Chaker Sfax, Sfax, Tunisia; 4 Laboratoire de Biochimie, Hôpital Habib Bourguiba Sfax, Sfax, Tunisia; 5 Département des Sciences de la vie, Faculté des Sciences de Sfax, Université des Sfax, Sfax, Tunisia

**Keywords:** Primary hyperoxaluria type 1, AGT, *AGXT* gene, Mutation, Compound heterozygous

## Abstract

**Background:**

Primary hyperoxaluria type 1 (PH1) is an autosomal recessive inherited disorder of glyoxylate metabolism in which excessive oxalates are formed by the liver and excreted by the kidneys. Calcium oxalate crystallizes in the urine, leading to urolithiasis, nephrocalcinosis, and consequent renal failure if treatment is not initiated promptly. Mutations in the *AGXT* gene which encodes the hepatic peroxisomal enzyme alanine:glyoxylate aminotransferase are responsible of PH1. In the present work, we aimed to analyze *AGXT* gene and in silico investigations performed in four patients with PH1 among two non consanguineous families.

**Methods:**

Exhaustive gene sequencing was performed after PCR amplification of coding exons and introns boundaries. Bioinformatic tools were used to predict the impact of *AGXT* variants on gene expression as well as on the protein structure and function.

**Results:**

Direct sequencing of all exons of *AGXT* gene revealed the emergence of multiple mutations in compound heterozygous state in the two studied families. Two patients were compound heterozygous for the c.731 T > C, c.32C > T, c.1020A > G and c.33_34insC and presented clinically with recurrent urinary tract infection, multiple urolithiasis and nephrocalcinosis under the age of 1 year and a persistent hyperoxaluria at the age of diagnosis. The two other patients presenting a less severe phenotypes were heterozygous for c.731 T > C and homozygous for the c.32C > T and c.1020A > G or compound heterozygous for c.26C > A and c.65A > G variants.

**Conclusion:**

In Summary, we provided relevance regarding the compound heterozygous mutations in non consanguineous PH1 families with variable severity.

## Background

Primary hyperoxalurias (PH) are a group of three autosomal recessive inherited disorders of glyoxylate metabolism [1]. PH1 (OMIM # 259900) represents the most common and severe type of primary hyperoxalurias, caused by mutations in the *AGXT* gene encoding the liver peroxisomal enzyme alanine:glyoxylate aminotransferase (AGT; EC 2.6.1.44) [2, 3]. The AGT deficiency in PH1 is responsible for an increased endogenous production and urinary excretion of oxalate that leads to hyperoxaluria. Calcium oxalate crystallizes in the urine, leading to recurrent urolithiasis, nephrocalcinosis and subsequently to renal failure and systemic oxalosis in the absence of appropriate early conservative treatment [4]. It is, therefore, necessary to establish a precise diagnosis early in the course of the disease to lead to an effective treatment.

An accurate PH1 diagnosis currently requires a measurement of AGT catalytic activity in the liver biopsy. Unfortunately, it is only available in a limited number of laboratories worldwide and it requires strict conditions for transport as the sample needs to be shipped frozen. These strict conditions make it difficult to PH1 patients to have liver biopsy and enzyme testing [5]. As an alternative approach, molecular analysis of *AGXT* gene allows a non invasive method to establish the PH1’s diagnosis in most of suspected patients. The *AGXT* gene, which encodes the 392 amino acid protein AGT, has been mapped to the 2q37.3 chromosome telomeric region. It is organized in 11 exons that spread across approximately 10 kb. Over 160 *AGXT* distinct mutations have so far been identified in PH1 as the cause of a wide spectrum of clinical severity (http://www.hgvs.org/) [6]. The most of mutant alleles (75%) are of single nucleotide substitutions type, particularly missense mutations. The remaining mutations are due to by either major or minor deletions and insertions. Functional studies showed that deficiency of AGT enzyme due to missense mutations could lead to decrease the AGT ability to fold correctly into active dimers or in some other cases to mitochondrial mistargeting of the enzyme instead to the hepatic peroxisomes.

In this study, we performed the *AGXT* gene molecular genetic analysis of in four Tunisian patients with PH1 syndrome. We highlighted the existence and severity of the compound heterozygous mutations in non consanguineous Tunisian families.

## Methods

### Patients and families

Four patients from two unrelated Tunisian families diagnosed with PH1 were studied. Their parents and healthy siblings were also subject to the same study.

The control group consisted of 50 healthy unrelated individuals with no family history of renal stones. Informed consent was obtained from both patients and control individuals in accordance with the ethics committee of Hedi Chaker Hospital (Sfax, Tunisia). The PH1 diagnosis was made based on clinical manifestation, biochemical and radiological data. Mean clinical characteristics observed in the studied patients were summarized in Table 1.

**Table 1:** Patients’ Baseline Demographics and key Clinical characteristics

Family	Patient	Sex	Age (years)	Consanguinity	Family History	Age of onset	Clinical and radiological presentation	Creat plas (μmol/l) Clearance (ml/mn/1,73m^2^)	Crystalluria	Oxaluria (μmol/24 h)	Vit B6 stable dose for at least 3 months	Progression to ESRD	Age of ESRD
Family F1	P 1	M	41	Yes	Yes	4 years	Recurrent urolithiasis			8609	No	Yes	41 years
	P 2	M	6	No	Yes	<1 year	Recurrent urinary tract infections, urolithiasis and Nephrocalcinosis	Creat: 28 Cl: 117	CaOx monohydrate crystals (type Ic whewellite). (500/mm^3^)	724.5	Yes (20 mg/24 h)	No	–
	P 3	M	4	No	Yes	<1 year	Recurrent urinary tract infections, urolithiasis and Nephrocalcinosis	Creat: 43 Cl: 73	CaOx monohydrate crystals (type Ic whewellite) (200/mm^3^).	599	Yes (20 mg/24 h)	No	–
Family F2	P 4	M	35	No	Yes (his sister was operated for renal lithiasis)	23 years	Recurrent urolithiasis and Nephrocalcinosis, operated for renal bilateral renal lithiasis for 5 times at ages 23, 24 and 25 years.	Creat: 236 (2012)	CaOx monohydrate crystals (type Ic whewellite). (250/mm^3^)	623	No	No	–

*Abbreviations*: *M* Male, *MD* Missing Data

### Molecular approach

#### DNA extraction

Blood samples from affected individuals and their family members were collected. Genomic DNA was extracted from leukocytes using the standard phenol-chloroform procedures.

#### PCR amplification and DNA sequencing

PCR amplification was performed for the 11 exons of the *AGXT* gene and their exon-intron boundary regions using appropriate primers. All exons were amplified in a thermal cycler (Applied Biosystem 2720) in a final volume of 50 μl containing 50 to 100 ng of genomic DNA, 0.2 μM of each primer, 1× PCR buffer (Promega), 1.2 mM MgCl2, 0.2 mM each dNTP, and 1 U Taq DNA polymerase (Promega, Madison, Wi, USA). PCR reactions were performed under the touchdown conditions as follows: initial denaturation at 95 °C for 5 min followed by 35 cycles of denaturation at 95 °C for 30 s, annealing at 65–58 °C (depending upon the primers sets used) for 30 s, extension at 72 °C for 45 s and final extension at 72 °C for 10 min.

The PCR products were then purified by enzyme reaction (Exonucléase I; 20 units/ μl; Fermentas), and directly sequenced on both strands using a Big-Dye v1.1 di-deoxy-terminator cycle sequencing kit on automated DNA sequencer ABI PRISM 3100-Avant (Perkin Elmer, Norwalk, CT, USA). The resulting sequences were aligned with reference sequences using the blast homology programs available at the NCBI (National Center for Biotechnology Information) website (https://blast.ncbi.nlm.nih.gov/Blast.cgi).

#### The sequence alignment

The sequence alignment of the *AGXT* gene was performed using the ClustalW program (http://www.ebi.ac.uk/Tools/msa/clustalo/).

#### Secondary structure analysis

The AGT protein secondary structure was obtained by NPS@ (Network Protein Sequence @nalyse). The secondary structure calculations were performed using by STRIDE (http://webclu.bio.wzw.tum.de/stride/). Prediction of the different variants effect on the mRNA secondary structure was performed using the MFOLD program.

#### Prediction of possible impact of an amino acid substitution

The possible impacts of the amino acid substitution on the three-dimensional protein structure, on the one hand, and that of the *AGXT* change in the protein function, on the other, were assessed using the PolyPhen program (Polymorphism Phenotyping) (http://genetics.bwh.harvard.edu/pph2/index.shtml).

The protein conformation was predicted applying by the program Deep View/Suiss-PDB viewer 3.7. (http://spdbv.vital-it.ch/) allowing the comparison of structures of both wild-type and mutant AGT subunits. Protein crystal structures were taken from PDB by 3D–JIGSAW. The models were evaluated on the basis of geometrical and stereochemical constraints using ProSA-Web (https://prosa.services.came.sbg.ac.at/prosa.php) and Verify 3D (http://services.mbi.ucla.edu/Verify_3D/).

## Results

### AGXT gene mutational analysis and Bioinformatic investigations

A mutational analysis of the 11 exons of *AGXT* gene and their flanking regions was performed in the studied patients and their parents in families F1 and F2. The results show several known variants and a novel one (Table 2).

**Table 2:** *AGXT* point variations detected in the studied patients with PH1

Family	Patient	Nucleotide change	Position	State	Codon/Effect	Ref
Family F1	P1	c.32C > T	Exon 1	Hom	p.Pro11Leu	Purdue et al., 1990
		Dup 74pb	Intron 1	Hom	Non coding	Purdue et al., 1991
		c.264C > T	Exon 2	Hom	p.Ala88Ala	Danpure et al., 1994
		c.358 + 13C > T	Intron 2	Hom	Non coding	Von Schnakenburg, 1998
		c.524 + 91C > T	Intron 4	Hom	Non coding	Monico et al., 2007
		c.680 + 17C > T	Intron 6	Hom	Non coding	Monico et al., 2007
		c.731 T > C	Exon 7	Het	p.Ile244Thr	Von Schnakenburg and Rumsby, 1997
		c.777-45C > T	Intron 7	Hom	Non coding	Von Schnakenburg, 1998
		c.777-44A > G	Intron 7	Hom	Non coding	Monico et al., 2007
		c.846 + 52G > A	Intron 8	Hom	Non coding	Monico et al., 2007
		c.1020A > G	Exon 10	Hom	p.Ile340Met	Purdue et al., 1992
		c.41C > A	3’UTR	Het	Non coding	Von Schnakenburg and Rumsby, 1997
	P2 and P3	c.32C > T	Exon 1	Het	p.Pro11Leu	Purdue et al., 1990
		c.33_34insC	Exon 1	Het	p.Lys12GlnfsX156	Pirilli et al., 1999
		Dup 74pb	Intron 1	Het	Non coding	Purdue et al., 1991
		c.264C > T	Exon 2	Het	p.Ala88Ala	Danpure et al., 1994
		c.358 + 13C > T	Intron 2	Het	Non coding	Von Schnakenburg, 1998
		c.524 + 91C > T	Intron 4	Het	Non coding	Monico et al., 2007
		c.680 + 17C > T	Intron 6	Het	Non coding	Monico et al., 2007
		c.731 T > C	Exon 7	Het	p.Ile244Thr	Von Schnakenburg and Rumsby, 1997
		c.777-45C > T	Intron 7	Het	Non coding	Von Schnakenburg, 1998
		c.777-44A > G	Intron 7	Het	Non coding	Monico et al., 2007
		c.846 + 52G > A	Intron 8	Het	Non coding	Monico et al., 2007
		c.1020A > G	Exon 10	Het	p.Ile340Met	Purdue et al., 1992
		c.41C > A	3’UTR	Het	Non coding	Von Schnakenburg and Rumsby, 1997
Family F2	Patient P4	c.26C > A	Exon 1	Het	p.Thr9Asn	Monico et al., 2005
		c.65A > G	Exon 1	Het	p.Asn22Ser	Williams et al., 2009
		c.524 + 91C > T	Intron 4	Het	Non coding	Monico et al., 2007
		c.777-44A > G	Intron 7	Hom	Non coding	Monico et al., 2007
		c.777-77A > G	Intron 7	het	Non coding	This paper
		c.41C > A	3’UTR	Het	Non coding	Von Schnakenburg and Rumsby, 1997

### AGXT mutation analysis in family F1

**Fig. 1:**
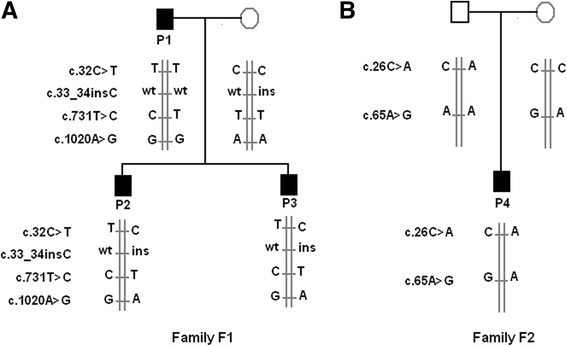
**a** Pedigree of the Family F1 showing the inheritance of the c.32C > T, c.731 T > C and c.33_34insC mutations in the affected members P1, P2 and P3. **b** Pedigree of the Family F2 showing the inheritance of the c.26C > A and c.65A > G variations in the affected members P4

**Fig. 2:**
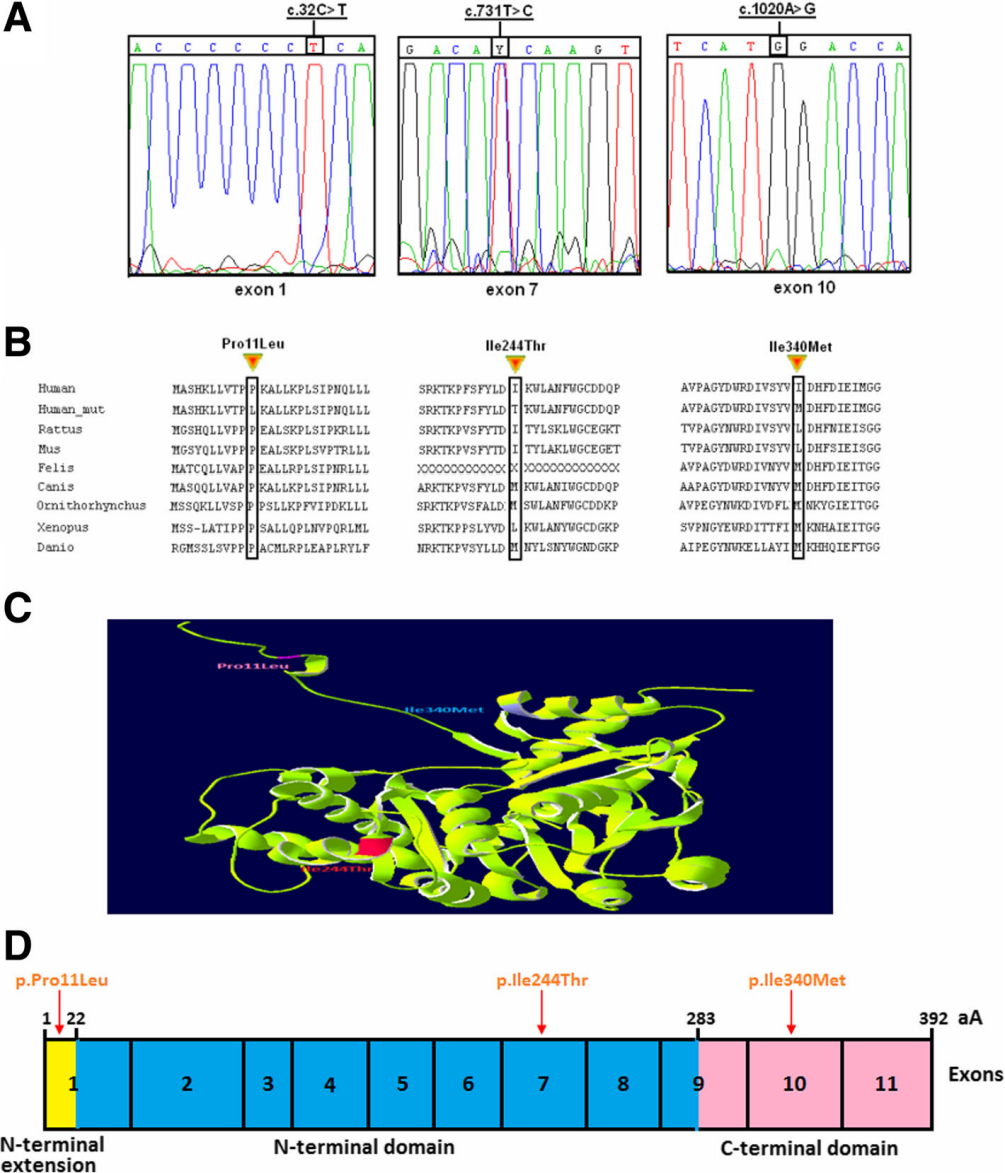
**a** Sequence chromatograms showing the presence of c.32C > T, c.731 T > C and c.1020A > G variants in Family F1. **b** Multiple sequence alignment of alanine:glyoxylate aminotransferase (AGT) with different species showing the aminoacids at position 11, 244 and 340. The concerned amino acids are boxed. **c** Model of AGT-LTM variant obtained by Deep View/Suiss-PDB viewer 3.7 software showing the aminoacids at position 11, 244 and 340. **d** A schematic drawing of the AGT domains showing the aminoacids at position 11, 244 and 340

In the non consanguineous family F1, more than one pathogenic known variants was detected: c.32C > T, c.731 T > C, c.33_34insC and c.1020A > G, together with other variants. In fact, the father (patient P1) was heterozygous for the c.731 T > C variant and homozygous for the c.32C > T and c.1020A > G variants whereas the unaffected mother was only heterozygous for the c.33_34insC. The two sons (patients P2 and P3) were compound heterozygous for the variants c.32C > T, c.731 T > C, c.1020A > G transmitted from the father and for c.33_34 insC transmitted from the mother (Figs. 1a and 2a).

The c.32C > T in exon 1, c.731 T > C in exon 7 and c.1020A > G in exon 10 were non synonymous variants leading to amino acid changes p.Pro11Leu, p.Ile244Thr and p.Ile340Met respectively (Fig. 2a) whereas c.33_34insC is a frameshift mutation occurring in exon 1 causing a premature termination codon at position 167 (TGA).

### Bioinformatic investigations of the detected variants

The bioinformatic tools were used to investigate the effect of the variants c.32C > T (p.Pro11Leu), c.731 T > C (p.Ile244Thr) and c.1020A > G (p.Ile340Met) and c.33_34insC on the generated AGT proteins.

The alignment of the AGT proteins in several species showed that a frameshift mutation c.33_34insC (p.Lys12GlnfsX156) occurred in the highly stable N-terminal extension of the AGT protein. It also revealed, that proline substituted by leucine in codon 11 and the isoleucine substituted by threonine in codon 244 affect a highly conserved nucleotide of the N-terminal extension of the AGT protein (residues 1–21) with a CI of 100% (Fig. 2b) and the large N-terminal domain (residues 22–282) respectively. However, the substitution of isoleucine 340 by a methionine is found in helix 331–344 in the small C-terminal domain (Fig. 2c and d).

The structural effect of these variants was analyzed using the NPS program. A secondary structure analysis of the modeled protein revealed that the wild type contained 27.81% alpha helix, 18.11% extended strand and 54.08% random coil. However, the mutant protein contained 32.91% alpha helix, 13.78% extended strand and 53.32% random coil. Our findings show that the main effect of these missense mutations was the introduction of new alpha helix into the amino acid chain. Furthermore, the secondary structure calculated by STRIDE shows that p.Pro11Leu, p.Ile244Thr and p.Ile340Met altered the backbone angles, respectively, at these positions (wild type: Phi ¼ ~ −58,31, Psi ¼ ~ 127,91; Phi ¼ ~ −59,41, Psi ¼ ~ −26,70; Phi ¼ ~ −73,23, Psi ¼ ~ −37,23; AGT-LTM: Phi ¼ ~ −57,52, Psi ¼ ~ 120,40; Phi ¼ ~ −58,03, Psi ¼ ~ −37,39; Phi ¼ ~ −66,97, Psi ¼ ~ −50,53), suggesting that these substitutions could destabilize the helical conformation of the peptide.

### AGXT mutation analysis in family F2 revealed two nucleotide changes in the AGT protein N-terminal region

The *AGXT* gene screening results in Patient 4 (P4) revealed the presence of known variants, summarized in Table 2. Two nucleotide changes in exon 1, c.26C > A and c.65A > G were found in heterozygous state and were inherited from each parent (Fig. 1b). The screening of these variants in 100 alleles from healthy unrelated individuals from the same region revealed their absence. The c.26C > A and c.65A > G result in two missense substitutions of threonine to asparagine at codon 9 (p.Thr9Asn) and asparagine to serine at codon 22 (p.Asn22Ser), respectively, in the N-terminal tail of AGT protein that grabs the other subunit within the dimer.

### Bioinformatic evaluation of the effect of c.26C > A and c.65A > G variants on RNA secondary structure and protein conformation

**Fig. 3:**
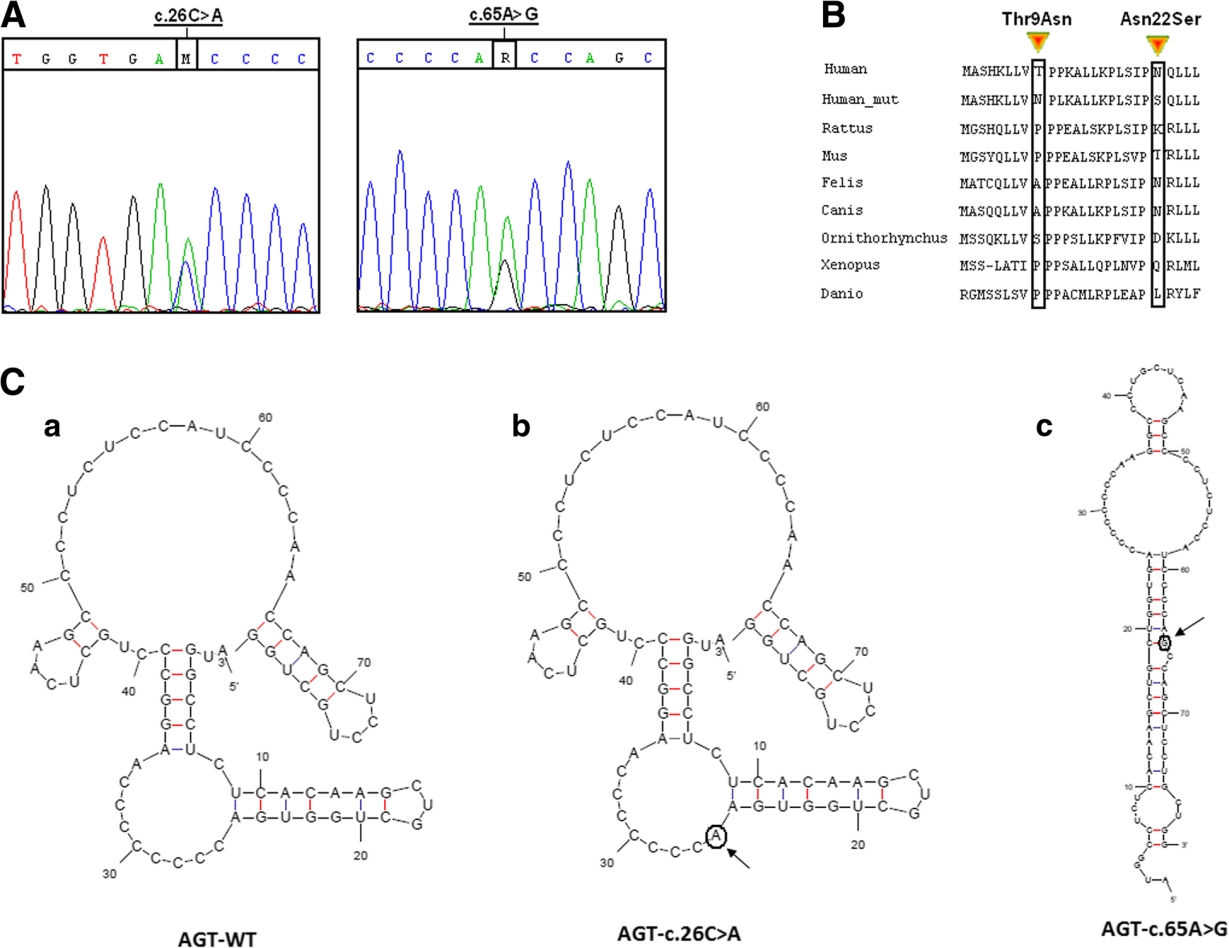
**A**) Sequence chromatograms showing the presence of c.26C > A and c.65A > G variants in Family F2. **B**) Multiple sequence alignment of alanine:glyoxylate aminotransferase (AGT) with different species showing the aminoacids at position 9 and 22. The concerned amino acids are boxed. **C**) Prediction of the effect of c.26C > A and c.65A > G variants on mRNA secondary structure; **a**) the wild-type AGT secondary structure; **b**) c.26C > A sowed no alteration on the RNA secondary structure; **c**) c.65 introduced several changes in the overall RNA secondary structure

The alignment of the AGT protein aminoacid sequences from different species revealed that the residues threonine and asparagine at codon position 9 and 22 respectively, are slightly conserved across the analyzed species (Fig. 3A, B). Besides, the evaluation of the effect of p.Thr9Asn and p.Asn22Ser on protein conformation showed some difference in the overall architecture of the mutant proteins compared to the human AGT wild-type. In fact, the r.m.s.deviation (root mean square deviation) for backbone atoms between the two structures was 0.2 Ǻ for p.Thr9Asn and 0.44 Ǻ for p.Asn22Ser.

The effect of c.26C > A and c.65A > G variants was then assessed using the MFOLD program. The results showed that the first would require virtually no alteration in the RNA secondary structure. However, the adenine substitution by guanine at position c.65 introduced several changes in the overall RNA secondary structure. In fact, the mutated sequence contained three hairpin loops between C^6^C^10^G^75^G^78^, A^25^G^36^C^50^U^59^ and G^36^C^48^ respectively. This new structure seemed to be widely different from the wild type structure in the number, composition and orientation of the hairpin loops. The wild type structure was formed essentially by two hairpin loops between A^1^C^66^G^79^ and U^7^U^9^A^25^A^35^ interrupted by three small ones between G^15^C^19^, C^43^G^48^ and C^70^G^75^ (Fig. 3C).

## Clinical severity evaluation of the compound heterozygous studied patients

The comparison of the clinical severity in the four PH1 studied patients was based on the patient’s age of onset, the clinical presentation and the disease outcome.

**Fig. 4:**
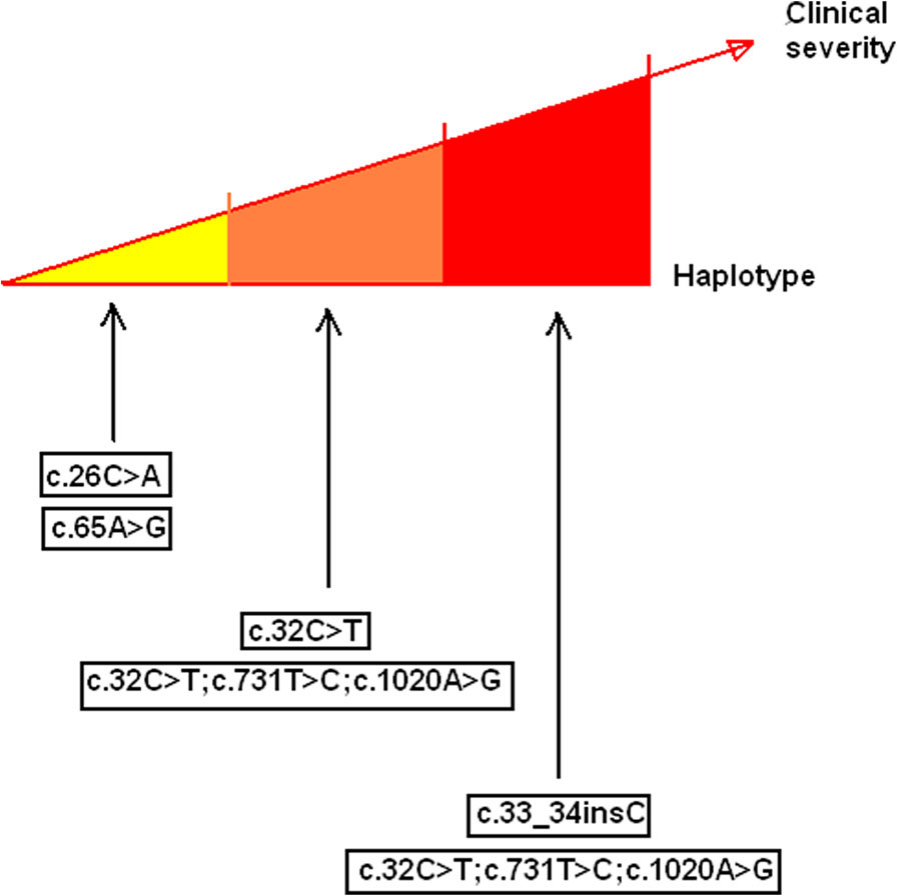
A schematic drawing of clinical severity evaluation of the studied patients with different haplotypes

The two patients (Patient P2 and P3), sharing the same haplotype: compound heterozygous for the c.731 T > C, c.32C > T, c.1020A > G and c.33_34insC showed the disease symptoms early with recurrent urinary tract infection, multiple urolithiasis and nephrocalcinosis within the first year after birth. These two cases presented a persistent hyperoxaluria at the time of diagnosis. The performed crystalluria in these two patients showed monohydrated calcium oxalate crystal, or whewellite, in the urine with a crystalline volume exceeding 200/mm^3^. Interestingly, P2 and P3 respond to pyridoxine treatment and thus their oxaluria values declined noticeably after three months of treatment. The Patients heterozygous for c.731 T > C and homozygous for the c.32C > T and c.1020A > G (Patient P1) and compound heterozygous for c.26C > A and c.65A > G (Patient P4) presented the first symptoms in childhood and in adulthood respectively. Patient P1 progressed to end stage renal disease at the age of 41 year old, whereas the renal function of the other patients is currently normal. The clinical presentation of Patient P4 seemed to be less severe with later age of onset and maintained renal function. The vitamin B6 palliative treatment with did not show any response for P1 and P4. A clinical severity evaluation of the studied patients is proposed in Fig. 4.

## Discussion

The present study reported clinical and molecular analysis of *AGXT* gene performed in four patients with PH1 among two non consanguineous families from Southern of Tunisia. Direct sequencing of all *AGXT* gene exons revealed the emergence of multiple mutations in compound heterozygous state in the two studied families.

In Tunisia, a country characterized by a high consanguinity rate, most of the reported cases belong to consanguineous families and were described with a single homozygous mutation [7–9]. In this report, more than one mutation was detected in the non consanguineous Family F1. The c.731 T > C mutation (p.Ile244Thr) in exon 7, c.32C > T mutation (p.Pro11Leu) in exon 1 and c.1020A > G (p. Ile340Met) in exon 10 were detected in patient P1 (the father) in the heterozygous state and in the homozygous state respectively. Patients 2 and 3 were compound heterozygous for the c.731 T > C, c.32C > T and c.1020A > G which they inherited from their father and for c.33_34insC in exon 1 inherited from their mother. In addition, patient 4 (P4), belonging to the non consanguineous Family F2, presented only two known nucleotide changes in exon 1, c.26C > A and c.65A > G in heterozygous state which he inherited from his father and the mother, respectively.

We have previously provided evidence regarding the potential involvement of c.32C > T, originally described as a common polymorphism, on PH1 phenotype [10]. The c.731 T > C mutation (p.Ile244Thr) and c.33_34insC mutation (p.Lys12GlnfsX156) have been reported as the two common causative mutations of PH1 in Tunisian patients with predominance of the c.731 T > C (p.Ile244Thr). Interestingly, in the present study, two patients were compound heterozygous for c.33_34insC mutation (p.Lys12GlnfsX156), c.32C > T (p.Pro11Leu), c.731 T > C (p.Ile244Thr) and c.1020A > G (p.Ile340Met) corresponding to AGT-LTM associated to p.Lys12GlnfsX156.

The functional studies confirmed that Ile244Thr mutation alone did not affect the AGT enzymatic activity [11, 12]. However, the coexistence of this mutation with c.32C > T (p.Pro11Leu) substitution causes a stable interaction between the AGT protein and molecular chaperones and thus leads to the aggregation and a rapid degradation of this enzyme [12]. The AGT activity was decreased to less than 5% in the presence of these two variants [12]. In addition, the c.33_34insC mutation leads to a frameshift and premature termination. Thus, a truncated protein of 167 instead of 392 amino acids for the wild one was generated. The homozygous c.33_34insC mutation biochemical phenotype was analyzed by previous studies [13]. The results showed that the liver biopsies of individuals presenting this mutation had no immunoreactive protein and no catalytic activity compared to other mutations due to peroxisome to mitochondrial mistargeting [13]. Several previous studies reported the presence of c.33_34insC in homozygous or compound heterozygous state with c.508G > A (p.Gly170Arg), c.698G > T (p.Arg233Leu), c.454 T > A (p.Phe152Ile), c.907C > T (p.Gln303X) and IVS8 + 1G > T splicing mutation [13–16].

The mutational analysis in patient 4 (P4) of the Family F2 showed the presence of reported variations c.26C > A (p.Thr9Asn) and c.65A > G (p.Asn22Ser) in a compound heterozygous state. The two variants c.26C > A (p.Thr9Asn) and c.65A > G (p.Asn22Ser) detected in the patient P4 at the heterozygous state, occurred in the N-terminal tail of AGT protein. The AGT homodimer folded into a large N-terminal domain, a smaller C-terminal domain and a 22 amino acid long unstructured N-terminal tail that grabs the other subunit within the dimer. This last region represents a highly conserved AGT subunit domain which has an important role in the dimerization and stabilization of the two subunits [17]. Indeed, Montioli and Cellini proved that artificial amino acid replacements in this extension create changes in the AGT functional properties in mammalian cells including redirection of the AGT enzyme from peroxisomes to mitochondria and that the N-terminal extension plays an essential role in allowing the AGT to reach its correct conformation and functional activity [18]. So the coexistence of these missense changes Thr9Asn and Asn22Ser in an important AGT protein region may explain the patient P4 phenotype and we may therefore hypothesize that these changes are of functional significance and may cause alterations in the protein structure.

The c.26C > A variation was reported for the very first time by Monico et al., in 2005 [19] and soon after, Monico et al., (2007) relying screened blood samples in a cohort of 55 unrelated probands with a conclusive diagnosis of PH1 [15]. In Monico’s et al. (2007) study, one patient was found to be in a homozygous state for c.26C > A. In fact, the c.26C > A variant was reported as a polymorphism [20] and also as a mutation [15] which is in accordance with our finding that revealed its absence in 100 normal alleles. The c.26C > A variant role as a common polymorphism was discussed relying only on sequence alignment data indicating that Thr at position 9 does not affect a highly conserved amino acid [17]. The evaluation of the effect of p.Thr9Asn on protein conformation of the mutant protein compared to the human AGT wild-type performed in this study, showed a difference in the overall architecture with an r.m.s.deviation (root mean square deviation) 0.2 Ǻ for p.Thr9Asn for backbone atoms compared to the wild type.

The second variant c.65A > G (p.Asn22Ser) had also been described as a common polymorphism with low frequency at 0.05 despite the lack of a molecular proof of its pathogenic effect [15]. In our research work, the evaluation of the effect of p.Asn22Ser on protein conformation showed some difference in the overall architecture of the mutant proteins compared to wild-type human AGT (rmsd = 0.44 Ǻ). The effect of c.65A > G variant was also assessed using the MFOLD program. Our results show that the substitution of the adenine by guanine at position c.65 introduced important changes in the overall RNA secondary structure. The mRNA folding was evidenced to influence a diverse range of transcription events such as pre mRNA splicing, processing, translational control and regulation [21]. These observations emphasize the possibility that c.65A > G variant can destabilize the generated transcript.

Several studies reported compound heterozygous cases in PH1 with severe symptoms [14, 22]. In our work, we reported three compound heterozygous patients belonging to two unrelated non consanguineous Tunisian families. The comparison of clinical severity in the PH1 studied compound heterozygous patients was based on the age of onset, the clinical presentation and the disease outcome and revealed the more severe phenotype in the patients sharing the same haplotype: compound heterozygous for the c.731 T > C, c.32C > T, c.1020A > G and c.33_34insC. The disease appeared the first year after birth with recurrent urinary tract infection, multiple urolithiasis and nephrocalcinosis with persistent hyperoxaluria at the age of diagnosis. This severity is correlated to the presence of two mutated alleles in compound heterozygous state leading to the production of two deficient proteins.

Patients P1 and P4 with less severe phenotypes, were heterozygous for c.731 T > C and homozygous for the c.32C > T and c.1020A > G or compound heterozygous for c.26C > A and c.65A > G. In comparison with other previous reports, Benhaj mbarek et al., reported on 57 Tunisian patients with PH1. In their cohort, genetic testing revealed 17 patients with Ile244Thr mutation and 8 carrying the c.33_34insC mutation. The median age at presentation for patients carrying the c.33_34insC mutation was 3 years (range 5 months-61 years), while those carrying the Ile244Thr mutation were older, with a median age of 13 years (range 3 months- 38 years) [8] . In the literature, several attempts have been made to establish a genotype-phenotype relationship for PH1 based on the disease age of onset. This disease onset age for homozygous of the three common mutations, c.33_34insC, c.508G > A and c.731 T > C was compared and showed that individuals homozygous for c.33_34insC were affected very early [13, 23, 24]. However, the late presentation of some patients was reported in several studies [10, 13, 16, 24]. In this study, the end stage renal disease was only observed in Patient 1 at the age of 41, whereas the renal function of the other patients has been normal so far. In the other reports, the clinical features of index cases vary from early-onset ESRD in the first year of life to recurrent or only occasional stone formation in adulthood. At the time of diagnosis, 20 to 50% of patients had advanced chronic kidney disease or even ESRD [3, 25]. An early diagnosis and an appropriate conservative management of this disease can delay kidney damage as log as possible.

## Conclusion

Primary hyperoxaluria is a rare metabolic disease characterized by allelic and clinical heterogeneity. In this study, we provided relevance regarding the compound heterozygous mutations in non consanguineous PH1 Tunisian families with a variable severity. The most severe phenotype was in patient in compound heterozygous state including c.731 T > C, c.32C > T, c.1020A > G and c.33_34insC mutation. In addition, two variations in exon 1 (c.26C > A (p.Thr9Asn) and c.65A > G (p.Asn22Ser)) were detected in a heterozygous state and described for the first time as the cause of PH1. The identification of the mutational spectrum of PH1 provides a rapid diagnosis and an appropriate management for affected patients.

## Abbreviations

AGT: Alanine:glyoxylate aminotransferase
PCR: Polymerase chain reaction
PH1: Primary hyperoxaluria type 1
rmsd: root mean square deviation
